# Crystal structure of [2-(tri­ethyl­ammonio)­eth­yl][(2,4,6-triiso­propyl­phen­yl)sulfon­yl]amide tetra­hydrate

**DOI:** 10.1107/S2056989015008105

**Published:** 2015-04-30

**Authors:** C. Golz, C. Strohmann

**Affiliations:** a Otto-Hahn-Strasse 6, Dortmund, D-44227, Germany

**Keywords:** crystal structure, zwitterion, hydrate, tape-like motif, hydrogen bonding

## Abstract

The zwitterionic title compound, C_23_H_42_N_2_O_2_S·4H_2_O, crystallized as a tetrahydrate from a solution of *N*-[(2,4,6-triiso­propyl­phen­yl)sulfon­yl]aziridine in tri­ethyl­amine, diethyl ether and pentane in the presence of moist air. It is formed by a nucleophillic ring-opening that is assumed to be reversible. The mol­ecular structure shows a major disorder of the triiso­propyl­phenyl group over two equally occupied locations. An inter­esting feature is the uncommon hydrate structure, exhibiting a tape-like motif which can be classified as a transition of the one-dimensional T4(2)6(2) motif into the two-dimensional L4(6)5(7)6(8) motif.

## Abstract 2

The zwitterionic title compound shows a major disorder of the triiso­propyl­phenyl group over two equally occupied locations. An inter­esting feature is the uncommon hydrate structure, exhibiting a tape-like motif which can be classified as a transition of the one-dimensional T4(2)6(2) motif into the two-dimensional L4(6)5(7)6(8) motif.

## Chemical context   

The title compound was isolated as by-product while purifying the corresponding sulfonyl­aziridine *via* column chromatography using a solvent mixture containing tri­ethyl­amine. Inter­estingly, the zwitterionic title compound was formed by the nucleophilic ring-opening of the aziridine. This is so far undocumented for tertiary amines but well known for primary or secondary amines (Hu, 2003 ▸). We assume that this ring-opening reaction is reversible, since the aziridine was isolated in the absence of water. Possibly, the zwitterionic structure is stabilized by the water mol­ecules and/or by crystallization, preventing the reverse reaction. Furthermore, the four incorporated solvent water mol­ecules in the crystal structure form a tape-like hydrate structure comparable to some known hydrogen-bonding motifs (Infantes *et al.*, 2003 ▸). This is discussed further in the *Supra­molecular features* section.
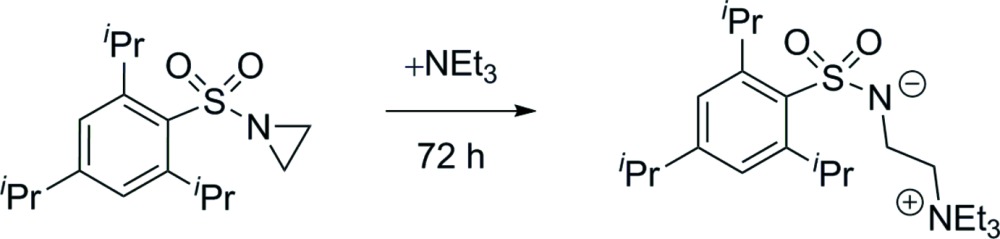


## Structural commentary   

**Figure 1:**
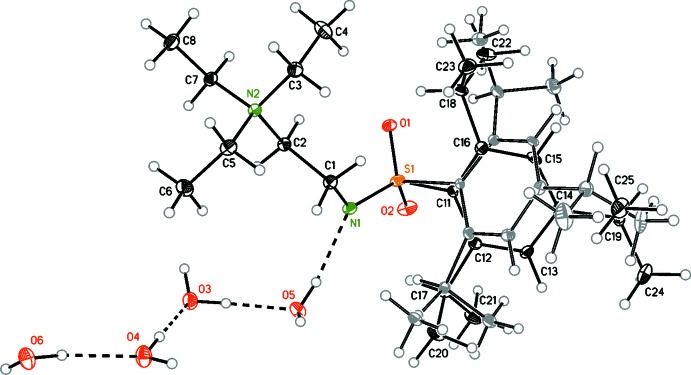
The mol­ecular structure and atom numbering for the title compound with displacement ellipsoids drawn at the 30% probability level. Atoms of the minor disorder component are drawn with grey-coloured C atoms.

The asymmetric unit consists of a [2-(tri­ethyl­ammonio)­eth­yl][(2,4,6-triiso­propyl­phen­yl)sulfon­yl]amide and four water mol­ecules (Fig. 1 ▸). The triiso­propyl­phenyl substituent is disordered over two slightly tilted locations with almost equal occupancies. No superlattice could be found and statistical disorder was assumed. Furthermore, the benzene ring appears to be bent towards the sulfur, which was also observed in the corresponding aziridine compound; for the structure of *rac*-2-phenyl-1-[(2,4,6-triiso­propyl­benzene)­sulfon­yl]aziridine, see Golz *et al.* (2014 ▸) and for isopropyl 2,4,6-triiso­propyl­phenyl sulfone see Sandrock *et al.* (2004 ▸). This seems to be typical of the triisoproyl­phenyl­sulfonyl group, though that will not be discussed further due to the disorder. The C2—N2 bond involving the cationic N atom is long [1.521 (2) Å], significantly exceeding the sum of the van der Waals radii (1.47 Å), while the C1—N1 bond [1.475 (2) Å], involving the anionic N atom, is close to the sum of the van der Waals radii. In contrast, the S—N1 bond [1.571 (1) Å] is shortened significantly, with the sum of the van der Waals radii being 1.73 Å. Both nitro­gen groups are in an almost perfect anti­periplanar conformation [N1—C1—C2—N2 = 179.7 (1)°].

## Supra­molecular features   

**Figure 2:**
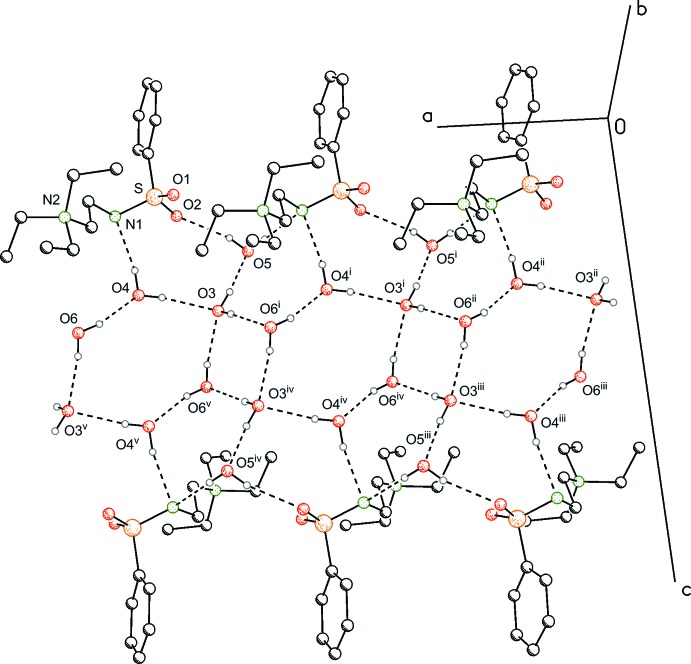
A view of the hydrate structure expanding along (100). H atoms not involved in hydrogen bonds and the isopropyl groups have been omitted for clarity. [Symmetry codes: (i) *x* − 1, *y*, *z*; (ii) *x* − 2, *y*, *z*; (iii) *x* − 1, *y* − 2, *z* − 1; (iv) *x* − 2, *y* − 2, *z* − 1; (v) *x* − 3, *y* − 2, *z* − 1.]

**Figure 3:**
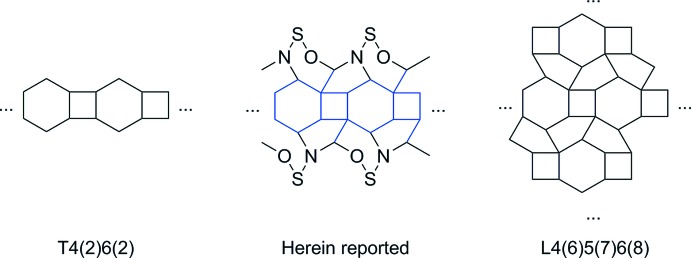
Hydrate-structure motifs already known (left and right) (Infantes *et al.*, 2003 ▸) and the structure reported here (centre).

Inter­molecular inter­actions occur mostly through hydrogen bonding of the water mol­ecules among themselves and with the zwitterionic compound (Table 1 ▸). Three of the four water mol­ecules form an infinite tape of inter­connected four- and six-membered rings known as the T4(2)6(2) motif. Each ring contains a centre of symmetry and the tape expands in the [100] direction. Inter­estingly, the border of the tape is lined with the zwitterionic compound and one additional water mol­ecule, thus expanding the tape with five- and six-membered rings involving the O4–O6–O3–O5–N1 and O4–O3–O5–O2–S1–N1 atoms, respectively (Fig. 2 ▸ and Fig. 3 ▸). The structure is comparable to the L4(6)5(7)6(8) motif, building up two-dimensional sheets, which are limited here by the zwitterionic amide. In summary, the hydrate structure discussed herein represents a transition between a one-dimensional tape and a two-dimensional sheet.

Some recent structures involving water forming the T4(2)6(2) hydrogen-bonding motif have been published (Li, Li, Su *et al.*, 2006 ▸; Li, Chen *et al.*, 2006 ▸; Song *et al.*, 2007 ▸; Kostakis *et al.*, 2009 ▸). There are only a few examples of two-dimensional hydrogen-bond networks known, but among these the L4(6)5(7)6(8) motif is the most common. For recent examples, see Born *et al.* (1995 ▸) and Gómez-Saiz *et al.* (2002 ▸).

## Database survey   

Comparable zwitterionic structures with neighbouring amide and ammonium groups are quite uncommon. Only one related structure was found in the Cambridge Structural database (Version 5.35, November 2013; Groom & Allen 2014 ▸). In the mol­ecule reported here, the N1—C1 bond length [1.475 (2) Å] involving the anionic N atom is normal [sum of van der Waals radii = 1.479 (2) Å], while the C2—N2 bond to the cationic N atom [1.521 (2) Å] is unusually long. This contrasts sharply with the structure of zwitterionic 1-amino-2-nitramino­ethane (Vasiliev *et al.*, 2001 ▸), where these observations are reversed, with the C—N bond to the anionic N atom reduced to 1.455 (2) Å.

## Synthesis and crystallization   

*N*-[(2,4,6-Triiso­propyl­phen­yl)sulfon­yl]aziridine was synthesized from ethano­lamine as described in the recent literature (Buckley *et al.*, 2013 ▸). Crystals of the title compound were obtained after a test tube containing small amounts of the sulfonyl­aziridine dissolved in a mixture of diethyl ether, pentane and tri­ethyl­amine was left to evaporate over a period of 3 d.

## Refinement   

Crystal data, data collection and structure refinement details are summarized in Table 2 ▸. All H atoms not involved in hydrogen bonds were positioned geometrically and refined using a riding model, with *U*_iso_(H) = 1.5*U*_eq_(C) for terminal and 1.2*U*_eq_(C) for non-terminal H atoms, with C—H = 0.98 Å. H atoms involved in hydrogen bonds were located in a difference Fourier synthesis map and were freely refined.

The disorder of the triiso­propyl­phenyl group was refined by a free variable to an occupancy ratio of 0.502 (2):0.498 (2). To ensure the stability of the phenyl ring in the refinement, the standard FLAT restraint was applied to atoms C11–C19 and a DELU restraint to atoms C11, C12 and C16, in both of the disorder domains. In addition, atoms C11, C11′ and C16′ required an additional ISOR restraint with a reduced deviation (s = 0.001 and st = 0.002).

## Supplementary Material

Crystal structure: contains datablock(s) I. DOI: 10.1107/S2056989015008105/sj5448sup1.cif

Structure factors: contains datablock(s) I. DOI: 10.1107/S2056989015008105/sj5448Isup2.hkl

Supporting information file. DOI: 10.1107/S2056989015008105/sj5448Isup3.cml

CCDC reference: 1061352

Additional supporting information:  crystallographic information; 3D view; checkCIF report

## Figures and Tables

**Table 1:** Hydrogen-bond geometry (, )

*D*H*A*	*D*H	H*A*	*D* *A*	*D*H*A*
O4H4*D*O3	0.83(2)	2.04(2)	2.867(2)	171(2)
O3H3*C*O5	0.90(2)	1.83(2)	2.725(2)	174(2)
O3H3*D*O6^i^	0.85(3)	2.08(3)	2.912(2)	169(2)
O5H5*C*O2^ii^	0.83(3)	2.09(3)	2.901(2)	165(2)
O6H6*D*O4	0.86(2)	1.95(2)	2.787(2)	167(2)
O6H6*E*O3^iii^	0.82(3)	2.03(3)	2.845(2)	170(2)
O5H5*D*N1	0.84(3)	2.05(3)	2.881(2)	170(2)
O4H4*E*N1^ii^	0.92(3)	2.06(3)	2.959(2)	165(3)

Symmetry codes: (i) 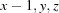; (ii) 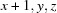; (iii) 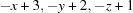.

**Table 2:** Experimental details

Crystal data	
Chemical formula	C_23_H_42_N_2_O_2_S4H_2_O
*M*_r_	482.71
Crystal system, space group	Triclinic, *P* 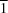
Temperature (K)	173
*a*, *b*, *c* ()	6.6797(4), 8.7345(5), 23.3973(14)
, , ()	96.579(5), 93.734(5), 95.570(5)
*V* (^3^)	1345.69(14)
*Z*	2
Radiation type	Mo *K*
(mm^1^)	0.16
Crystal size (mm)	0.34 0.25 0.08

Data collection	
Diffractometer	Agilent Xcalibur Sapphire3
Absorption correction	Multi-scan (*CrysAlis PRO*; Oxford Diffraction, 2013 ▸)
*T*_min_, *T*_max_	0.981, 1.000
No. of measured, independent and observed [*I* > 2(*I*)] reflections	34730, 5881, 4239
*R*_int_	0.075
(sin /)_max_ (^1^)	0.639

Refinement	
*R*[*F* ^2^ > 2(*F* ^2^)], *wR*(*F* ^2^), *S*	0.049, 0.102, 1.01
No. of reflections	5881
No. of parameters	472
No. of restraints	36
H-atom treatment	H atoms treated by a mixture of independent and constrained refinement
_max_, _min_ (e ^3^)	0.24, 0.33

Computer programs: *CrysAlis PRO* (Oxford Diffraction, 2013 ▸), *SHELXS97* and *SHELXL97* (Sheldrick, 2008 ▸), *OLEX2* (Dolomanov *et al.*, 2009 ▸) and *publCIF* (Westrip, 2010 ▸).

## supplementary crystallographic information

### Crystal data

**Table:**

C_23_H_42_N_2_O_2_S·4H_2_O	*Z* = 2
*M**_r_* = 482.71	*F*(000) = 532
Triclinic, *P*1	*D*_x_ = 1.191 Mg m^−^^3^
*a* = 6.6797 (4) Å	Mo *K*α radiation, λ = 0.71073 Å
*b* = 8.7345 (5) Å	Cell parameters from 5123 reflections
*c* = 23.3973 (14) Å	θ = 2.6–28.2°
α = 96.579 (5)°	µ = 0.16 mm^−^^1^
β = 93.734 (5)°	*T* = 173 K
γ = 95.570 (5)°	Plate, clear colourless
*V* = 1345.69 (14) Å^3^	0.34 × 0.25 × 0.08 mm

### Data collection

**Table:**

Agilent Xcalibur Sapphire3 diffractometer	5881 independent reflections
Radiation source: Enhance (Mo) X-ray Source	4239 reflections with *I* > 2σ(*I*)
Graphite monochromator	*R*_int_ = 0.075
Detector resolution: 16.0560 pixels mm^-1^	θ_max_ = 27.0°, θ_min_ = 2.4°
ω scans	*h* = −8→8
Absorption correction: multi-scan (*CrysAlis PRO*; Oxford Diffraction, 2013)	*k* = −11→11
*T*_min_ = 0.981, *T*_max_ = 1.000	*l* = −29→29
34730 measured reflections	

### Refinement

**Table:**

Refinement on *F*^2^	Primary atom site location: structure-invariant direct methods
Least-squares matrix: full	Hydrogen site location: mixed
*R*[*F*^2^ > 2σ(*F*^2^)] = 0.049	H atoms treated by a mixture of independent and constrained refinement
*wR*(*F*^2^) = 0.102	*w* = 1/[σ^2^(*F*_o_^2^) + (0.0428*P*)^2^ + 0.1372*P*] where *P* = (*F*_o_^2^ + 2*F*_c_^2^)/3
*S* = 1.01	(Δ/σ)_max_ = 0.001
5881 reflections	Δρ_max_ = 0.24 e Å^−^^3^
472 parameters	Δρ_min_ = −0.33 e Å^−^^3^
36 restraints	

### Special details

**Table:**

Geometry. All e.s.d.'s (except the e.s.d. in the dihedral angle between two l.s. planes) are estimated using the full covariance matrix. The cell e.s.d.'s are taken into account individually in the estimation of e.s.d.'s in distances, angles and torsion angles; correlations between e.s.d.'s in cell parameters are only used when they are defined by crystal symmetry. An approximate (isotropic) treatment of cell e.s.d.'s is used for estimating e.s.d.'s involving l.s. planes.

### Fractional atomic coordinates and isotropic or equivalent isotropic displacement parameters (Å^2^)

**Table:**

	*x*	*y*	*z*	*U*_iso_*/*U*_eq_	Occ. (<1)
S1	0.46930 (6)	0.84811 (5)	0.73798 (2)	0.01893 (12)	
O1	0.32141 (17)	0.72141 (14)	0.71224 (5)	0.0225 (3)	
O2	0.39889 (18)	1.00090 (14)	0.73798 (6)	0.0295 (3)	
O3	1.1266 (2)	1.00237 (18)	0.57740 (7)	0.0300 (3)	
O4	1.5372 (2)	0.93656 (17)	0.59417 (7)	0.0322 (4)	
O5	1.0118 (2)	1.06162 (16)	0.68669 (6)	0.0262 (3)	
O6	1.7836 (2)	0.81480 (17)	0.51262 (7)	0.0312 (4)	
N1	0.6732 (2)	0.85102 (16)	0.70803 (6)	0.0186 (3)	
C1	0.7592 (3)	0.70129 (19)	0.70154 (7)	0.0186 (4)	
H1A	0.7122	0.6373	0.7313	0.022*	
H1B	0.9083	0.7186	0.7062	0.022*	
N2	0.7612 (2)	0.46079 (15)	0.62443 (6)	0.0155 (3)	
C2	0.6901 (2)	0.61873 (18)	0.64150 (7)	0.0164 (4)	
H2A	0.5408	0.6063	0.6379	0.020*	
H2B	0.7347	0.6877	0.6130	0.020*	
C3	0.6882 (3)	0.3436 (2)	0.66417 (8)	0.0216 (4)	
H3A	0.7416	0.2440	0.6522	0.026*	
H3B	0.7462	0.3807	0.7039	0.026*	
C4	0.4624 (3)	0.3141 (2)	0.66513 (9)	0.0320 (5)	
H4A	0.4073	0.4116	0.6774	0.048*	
H4B	0.4298	0.2395	0.6922	0.048*	
H4C	0.4032	0.2722	0.6264	0.048*	
C5	0.9904 (2)	0.4708 (2)	0.62907 (8)	0.0213 (4)	
H5A	1.0404	0.5092	0.6694	0.026*	
H5B	1.0311	0.3652	0.6203	0.026*	
C6	1.0912 (3)	0.5746 (2)	0.58966 (8)	0.0291 (5)	
H6A	1.0489	0.5345	0.5494	0.044*	
H6B	1.2380	0.5770	0.5960	0.044*	
H6C	1.0522	0.6798	0.5980	0.044*	
C7	0.6761 (3)	0.4100 (2)	0.56254 (7)	0.0198 (4)	
H7A	0.5270	0.4016	0.5615	0.024*	
H7B	0.7194	0.4918	0.5386	0.024*	
C8	0.7380 (3)	0.2575 (2)	0.53547 (8)	0.0281 (5)	
H8A	0.8850	0.2656	0.5344	0.042*	
H8B	0.6745	0.2333	0.4961	0.042*	
H8C	0.6946	0.1750	0.5584	0.042*	
C11	0.5230 (8)	0.8325 (6)	0.8086 (2)	0.0128 (14)	0.5020 (15)
C12	0.6299 (5)	0.9594 (4)	0.84523 (15)	0.0160 (7)	0.5020 (15)
C13	0.6466 (5)	0.9557 (4)	0.90455 (14)	0.0179 (8)	0.5020 (15)
H13	0.7179	1.0416	0.9285	0.021*	0.5020 (15)
C14	0.5630 (5)	0.8313 (4)	0.93024 (14)	0.0171 (8)	0.5020 (15)
C15	0.4656 (9)	0.7066 (5)	0.8933 (3)	0.0181 (11)	0.5020 (15)
H15	0.4075	0.6203	0.9100	0.022*	0.5020 (15)
C16	0.4474 (9)	0.6995 (8)	0.8334 (3)	0.0168 (12)	0.5020 (15)
C17	0.7393 (5)	1.1005 (4)	0.82250 (15)	0.0172 (8)	0.5020 (15)
H17	0.7186	1.0852	0.7794	0.021*	0.5020 (15)
C18	0.3527 (5)	0.5456 (4)	0.80066 (16)	0.0203 (8)	0.5020 (15)
H18	0.3777	0.5485	0.7591	0.024*	0.5020 (15)
C19	0.5849 (5)	0.8221 (4)	0.99530 (15)	0.0218 (8)	0.5020 (15)
H19	0.4485	0.7879	1.0074	0.026*	0.5020 (15)
C20	0.9656 (5)	1.1076 (5)	0.83905 (17)	0.0293 (10)	0.5020 (15)
H20A	0.9905	1.1284	0.8811	0.044*	0.5020 (15)
H20B	1.0375	1.1906	0.8211	0.044*	0.5020 (15)
H20C	1.0139	1.0083	0.8255	0.044*	0.5020 (15)
C21	0.6519 (6)	1.2506 (4)	0.84431 (19)	0.0331 (10)	0.5020 (15)
H21A	0.5071	1.2408	0.8328	0.050*	0.5020 (15)
H21B	0.7197	1.3375	0.8276	0.050*	0.5020 (15)
H21C	0.6731	1.2693	0.8865	0.050*	0.5020 (15)
C22	0.1266 (10)	0.5259 (6)	0.8042 (3)	0.0256 (14)	0.5020 (15)
H22A	0.0962	0.5164	0.8441	0.038*	0.5020 (15)
H22B	0.0684	0.4322	0.7792	0.038*	0.5020 (15)
H22C	0.0686	0.6163	0.7914	0.038*	0.5020 (15)
C23	0.4510 (6)	0.4075 (4)	0.82033 (17)	0.0281 (9)	0.5020 (15)
H23A	0.5970	0.4234	0.8172	0.042*	0.5020 (15)
H23B	0.3947	0.3125	0.7958	0.042*	0.5020 (15)
H23C	0.4243	0.3980	0.8605	0.042*	0.5020 (15)
C24	0.6614 (6)	0.9742 (4)	1.03185 (16)	0.0297 (9)	0.5020 (15)
H24A	0.6546	0.9616	1.0728	0.045*	0.5020 (15)
H24B	0.5775	1.0549	1.0221	0.045*	0.5020 (15)
H24C	0.8015	1.0042	1.0243	0.045*	0.5020 (15)
C25	0.7232 (11)	0.6981 (7)	1.0074 (3)	0.0307 (14)	0.5020 (15)
H25A	0.8594	0.7299	0.9969	0.046*	0.5020 (15)
H25B	0.6711	0.5996	0.9845	0.046*	0.5020 (15)
H25C	0.7278	0.6854	1.0485	0.046*	0.5020 (15)
C11'	0.5178 (9)	0.8031 (6)	0.8164 (2)	0.0110 (13)	0.4980 (15)
C16'	0.3975 (9)	0.6931 (8)	0.8416 (3)	0.0122 (15)	0.4980 (15)
C12'	0.7007 (5)	0.8740 (4)	0.84657 (14)	0.0149 (7)	0.4980 (15)
C13'	0.7689 (5)	0.8181 (4)	0.89710 (15)	0.0175 (8)	0.4980 (15)
H13'	0.8919	0.8650	0.9171	0.021*	0.4980 (15)
C14'	0.6617 (5)	0.6952 (4)	0.91930 (14)	0.0165 (8)	0.4980 (15)
C15'	0.4741 (8)	0.6404 (5)	0.8922 (2)	0.0129 (10)	0.4980 (15)
H15'	0.3936	0.5636	0.9086	0.015*	0.4980 (15)
C17'	0.8201 (5)	1.0221 (4)	0.83205 (14)	0.0149 (7)	0.4980 (15)
H17'	0.7618	1.0467	0.7941	0.018*	0.4980 (15)
C18'	0.1755 (5)	0.6347 (5)	0.82125 (16)	0.0201 (8)	0.4980 (15)
H18'	0.1335	0.6925	0.7887	0.024*	0.4980 (15)
C19'	0.7393 (6)	0.6336 (5)	0.97372 (18)	0.0202 (8)	0.4980 (15)
H19'	0.6572	0.5330	0.9764	0.024*	0.4980 (15)
C20'	1.0444 (5)	1.0021 (4)	0.82672 (18)	0.0213 (8)	0.4980 (15)
H20D	1.1134	1.0970	0.8155	0.032*	0.4980 (15)
H20E	1.0571	0.9148	0.7974	0.032*	0.4980 (15)
H20F	1.1056	0.9817	0.8639	0.032*	0.4980 (15)
C21'	0.7914 (6)	1.1560 (4)	0.87833 (16)	0.0231 (8)	0.4980 (15)
H21D	0.8426	1.1322	0.9162	0.035*	0.4980 (15)
H21E	0.6476	1.1696	0.8790	0.035*	0.4980 (15)
H21F	0.8657	1.2516	0.8694	0.035*	0.4980 (15)
C22'	0.0387 (5)	0.6696 (5)	0.87050 (16)	0.0266 (9)	0.4980 (15)
H22D	−0.1029	0.6520	0.8552	0.040*	0.4980 (15)
H22E	0.0701	0.7778	0.8875	0.040*	0.4980 (15)
H22F	0.0618	0.6011	0.9001	0.040*	0.4980 (15)
C23'	0.1484 (11)	0.4617 (6)	0.7993 (3)	0.0250 (13)	0.4980 (15)
H23D	0.1894	0.4026	0.8305	0.037*	0.4980 (15)
H23E	0.2321	0.4417	0.7669	0.037*	0.4980 (15)
H23F	0.0064	0.4298	0.7864	0.037*	0.4980 (15)
C24'	0.7064 (13)	0.7460 (9)	1.0273 (3)	0.0349 (15)	0.4980 (15)
H24D	0.7516	0.7031	1.0622	0.052*	0.4980 (15)
H24E	0.5627	0.7599	1.0280	0.052*	0.4980 (15)
H24F	0.7840	0.8463	1.0257	0.052*	0.4980 (15)
C25'	0.9588 (6)	0.6021 (5)	0.97315 (18)	0.0364 (11)	0.4980 (15)
H25D	0.9743	0.5244	0.9405	0.055*	0.4980 (15)
H25E	1.0009	0.5633	1.0092	0.055*	0.4980 (15)
H25F	1.0428	0.6982	0.9694	0.055*	0.4980 (15)
H4D	1.414 (3)	0.946 (2)	0.5910 (9)	0.036 (7)*	
H3C	1.081 (3)	1.025 (3)	0.6124 (11)	0.048 (7)*	
H3D	1.036 (4)	0.937 (3)	0.5591 (12)	0.065 (9)*	
H5C	1.110 (4)	1.033 (3)	0.7046 (10)	0.049 (8)*	
H6D	1.695 (4)	0.853 (3)	0.5332 (10)	0.048 (7)*	
H6E	1.794 (4)	0.866 (3)	0.4853 (11)	0.056 (9)*	
H5D	0.909 (4)	1.010 (3)	0.6956 (11)	0.065 (9)*	
H4E	1.565 (4)	0.923 (3)	0.6322 (14)	0.094 (11)*	

### Atomic displacement parameters (Å^2^)

**Table:**

	*U*^11^	*U*^22^	*U*^33^	*U*^12^	*U*^13^	*U*^23^
S1	0.0136 (2)	0.0180 (2)	0.0234 (2)	0.00158 (17)	−0.00203 (18)	−0.00337 (18)
O1	0.0173 (6)	0.0259 (7)	0.0210 (7)	−0.0049 (5)	−0.0018 (5)	−0.0027 (5)
O2	0.0202 (7)	0.0226 (7)	0.0441 (9)	0.0081 (6)	−0.0053 (6)	−0.0041 (6)
O3	0.0257 (8)	0.0411 (9)	0.0229 (8)	0.0026 (7)	0.0028 (7)	0.0030 (7)
O4	0.0290 (9)	0.0445 (9)	0.0259 (8)	0.0138 (7)	0.0042 (7)	0.0067 (7)
O5	0.0211 (7)	0.0311 (8)	0.0278 (8)	0.0013 (6)	0.0031 (6)	0.0106 (6)
O6	0.0274 (8)	0.0390 (9)	0.0310 (9)	0.0115 (7)	0.0086 (7)	0.0103 (7)
N1	0.0162 (7)	0.0162 (7)	0.0225 (8)	0.0018 (6)	0.0007 (6)	−0.0012 (6)
C1	0.0165 (9)	0.0195 (9)	0.0196 (9)	0.0031 (7)	0.0001 (7)	0.0010 (7)
N2	0.0138 (7)	0.0157 (7)	0.0168 (8)	0.0030 (6)	0.0000 (6)	0.0008 (6)
C2	0.0167 (9)	0.0151 (9)	0.0180 (9)	0.0040 (7)	0.0014 (7)	0.0024 (7)
C3	0.0273 (10)	0.0165 (9)	0.0223 (10)	0.0042 (8)	0.0034 (8)	0.0050 (7)
C4	0.0281 (11)	0.0324 (11)	0.0382 (12)	0.0005 (9)	0.0113 (9)	0.0125 (9)
C5	0.0133 (9)	0.0240 (10)	0.0257 (10)	0.0055 (7)	−0.0027 (8)	−0.0011 (8)
C6	0.0157 (9)	0.0426 (12)	0.0270 (11)	−0.0029 (9)	−0.0011 (8)	0.0025 (9)
C7	0.0153 (9)	0.0247 (10)	0.0173 (9)	−0.0005 (7)	−0.0009 (7)	−0.0028 (7)
C8	0.0256 (10)	0.0279 (10)	0.0281 (11)	0.0022 (8)	0.0035 (9)	−0.0080 (9)
C11	0.0123 (16)	0.0136 (16)	0.0127 (16)	0.0009 (9)	0.0033 (9)	0.0017 (9)
C12	0.0134 (17)	0.0168 (19)	0.0178 (19)	0.0013 (15)	0.0003 (14)	0.0027 (15)
C13	0.0161 (17)	0.0200 (18)	0.0156 (18)	0.0007 (14)	−0.0019 (14)	−0.0033 (14)
C14	0.0153 (17)	0.0248 (19)	0.0129 (17)	0.0077 (14)	0.0028 (14)	0.0037 (15)
C15	0.020 (2)	0.011 (2)	0.025 (2)	0.000 (2)	0.0075 (17)	0.008 (3)
C16	0.013 (3)	0.020 (3)	0.018 (3)	0.003 (2)	0.002 (2)	−0.0006 (19)
C17	0.0174 (19)	0.0157 (19)	0.0170 (19)	−0.0038 (15)	0.0005 (15)	0.0003 (15)
C18	0.0231 (19)	0.0165 (18)	0.0203 (19)	−0.0058 (15)	−0.0003 (15)	0.0057 (15)
C19	0.0206 (19)	0.030 (2)	0.0155 (18)	0.0016 (16)	0.0041 (15)	0.0043 (16)
C20	0.019 (2)	0.038 (2)	0.031 (2)	−0.0049 (19)	−0.0003 (17)	0.0127 (19)
C21	0.036 (2)	0.019 (2)	0.046 (3)	0.0010 (17)	0.013 (2)	0.0055 (18)
C22	0.023 (3)	0.018 (4)	0.035 (3)	−0.003 (3)	−0.009 (2)	0.009 (3)
C23	0.038 (2)	0.0172 (19)	0.028 (2)	0.0010 (17)	0.0011 (18)	0.0013 (16)
C24	0.039 (2)	0.035 (2)	0.0153 (19)	0.0106 (19)	−0.0028 (17)	−0.0014 (17)
C25	0.045 (3)	0.026 (4)	0.022 (4)	0.008 (3)	−0.006 (3)	0.008 (3)
C11'	0.0113 (15)	0.0120 (16)	0.0097 (15)	0.0002 (9)	0.0018 (9)	0.0016 (9)
C16'	0.0128 (18)	0.0110 (16)	0.0132 (17)	0.0016 (10)	0.0013 (10)	0.0022 (9)
C12'	0.0190 (18)	0.0113 (18)	0.0152 (18)	0.0029 (15)	0.0066 (14)	0.0008 (14)
C13'	0.0144 (17)	0.0195 (18)	0.0176 (18)	−0.0010 (14)	−0.0005 (14)	0.0013 (14)
C14'	0.0174 (18)	0.0186 (18)	0.0136 (17)	0.0035 (14)	0.0013 (14)	0.0007 (14)
C15'	0.013 (2)	0.011 (2)	0.014 (2)	−0.003 (2)	0.0009 (15)	0.005 (2)
C17'	0.0159 (18)	0.0177 (19)	0.0104 (17)	−0.0023 (16)	−0.0014 (14)	0.0040 (15)
C18'	0.0189 (19)	0.023 (2)	0.0183 (19)	−0.0041 (16)	0.0020 (15)	0.0064 (17)
C19'	0.023 (2)	0.021 (2)	0.017 (2)	0.0015 (16)	−0.0015 (17)	0.0060 (17)
C20'	0.0162 (18)	0.023 (2)	0.024 (2)	−0.0024 (16)	0.0063 (16)	0.0035 (16)
C21'	0.026 (2)	0.0180 (18)	0.025 (2)	−0.0012 (15)	0.0074 (16)	0.0033 (16)
C22'	0.0156 (18)	0.037 (2)	0.028 (2)	0.0002 (16)	0.0021 (16)	0.0072 (18)
C23'	0.026 (3)	0.022 (3)	0.026 (3)	−0.004 (3)	0.0007 (19)	0.002 (3)
C24'	0.045 (3)	0.049 (5)	0.014 (3)	0.016 (3)	0.006 (3)	0.005 (3)
C25'	0.034 (2)	0.054 (3)	0.027 (2)	0.022 (2)	0.0028 (19)	0.018 (2)

### Geometric parameters (Å, º)

**Table:**

S1—O1	1.4563 (12)	C19—C24	1.520 (5)
S1—O2	1.4574 (13)	C19—C25	1.529 (7)
S1—N1	1.5708 (14)	C20—H20A	0.9800
S1—C11	1.692 (6)	C20—H20B	0.9800
S1—C11'	1.934 (6)	C20—H20C	0.9800
O3—H3C	0.90 (2)	C21—H21A	0.9800
O3—H3D	0.85 (3)	C21—H21B	0.9800
O4—H4D	0.83 (2)	C21—H21C	0.9800
O4—H4E	0.92 (3)	C22—H22A	0.9800
O5—H5C	0.83 (3)	C22—H22B	0.9800
O5—H5D	0.84 (3)	C22—H22C	0.9800
O6—H6D	0.86 (2)	C23—H23A	0.9800
O6—H6E	0.82 (3)	C23—H23B	0.9800
N1—C1	1.475 (2)	C23—H23C	0.9800
C1—H1A	0.9900	C24—H24A	0.9800
C1—H1B	0.9900	C24—H24B	0.9800
C1—C2	1.525 (2)	C24—H24C	0.9800
N2—C2	1.521 (2)	C25—H25A	0.9800
N2—C3	1.527 (2)	C25—H25B	0.9800
N2—C5	1.521 (2)	C25—H25C	0.9800
N2—C7	1.521 (2)	C11'—C16'	1.399 (8)
C2—H2A	0.9900	C11'—C12'	1.419 (7)
C2—H2B	0.9900	C16'—C15'	1.403 (9)
C3—H3A	0.9900	C16'—C18'	1.546 (7)
C3—H3B	0.9900	C12'—C13'	1.396 (5)
C3—C4	1.508 (2)	C12'—C17'	1.536 (5)
C4—H4A	0.9800	C13'—H13'	0.9500
C4—H4B	0.9800	C13'—C14'	1.401 (5)
C4—H4C	0.9800	C14'—C15'	1.383 (7)
C5—H5A	0.9900	C14'—C19'	1.518 (5)
C5—H5B	0.9900	C15'—H15'	0.9500
C5—C6	1.510 (3)	C17'—H17'	1.0000
C6—H6A	0.9800	C17'—C20'	1.536 (5)
C6—H6B	0.9800	C17'—C21'	1.535 (5)
C6—H6C	0.9800	C18'—H18'	1.0000
C7—H7A	0.9900	C18'—C22'	1.538 (5)
C7—H7B	0.9900	C18'—C23'	1.529 (5)
C7—C8	1.514 (2)	C19'—H19'	1.0000
C8—H8A	0.9800	C19'—C24'	1.542 (6)
C8—H8B	0.9800	C19'—C25'	1.518 (5)
C8—H8C	0.9800	C20'—H20D	0.9800
C11—C12	1.422 (6)	C20'—H20E	0.9800
C11—C16	1.420 (8)	C20'—H20F	0.9800
C12—C13	1.390 (5)	C21'—H21D	0.9800
C12—C17	1.537 (5)	C21'—H21E	0.9800
C13—H13	0.9500	C21'—H21F	0.9800
C13—C14	1.389 (5)	C22'—H22D	0.9800
C14—C15	1.390 (6)	C22'—H22E	0.9800
C14—C19	1.532 (5)	C22'—H22F	0.9800
C15—H15	0.9500	C23'—H23D	0.9800
C15—C16	1.392 (9)	C23'—H23E	0.9800
C16—C18	1.526 (7)	C23'—H23F	0.9800
C17—H17	1.0000	C24'—H24D	0.9800
C17—C20	1.529 (5)	C24'—H24E	0.9800
C17—C21	1.534 (5)	C24'—H24F	0.9800
C18—H18	1.0000	C25'—H25D	0.9800
C18—C22	1.512 (7)	C25'—H25E	0.9800
C18—C23	1.530 (5)	C25'—H25F	0.9800
C19—H19	1.0000		
			
O1—S1—O2	113.94 (7)	C17—C20—H20C	109.5
O1—S1—N1	112.66 (7)	H20A—C20—H20B	109.5
O1—S1—C11	110.2 (2)	H20A—C20—H20C	109.5
O1—S1—C11'	103.48 (18)	H20B—C20—H20C	109.5
O2—S1—N1	107.70 (8)	C17—C21—H21A	109.5
O2—S1—C11	104.22 (18)	C17—C21—H21B	109.5
O2—S1—C11'	109.94 (16)	C17—C21—H21C	109.5
N1—S1—C11	107.6 (2)	H21A—C21—H21B	109.5
N1—S1—C11'	108.99 (18)	H21A—C21—H21C	109.5
H3C—O3—H3D	105 (2)	H21B—C21—H21C	109.5
H4D—O4—H4E	105 (2)	C18—C22—H22A	109.5
H5C—O5—H5D	107 (2)	C18—C22—H22B	109.5
H6D—O6—H6E	107 (2)	C18—C22—H22C	109.5
C1—N1—S1	114.54 (11)	H22A—C22—H22B	109.5
N1—C1—H1A	110.1	H22A—C22—H22C	109.5
N1—C1—H1B	110.1	H22B—C22—H22C	109.5
N1—C1—C2	108.09 (14)	C18—C23—H23A	109.5
H1A—C1—H1B	108.4	C18—C23—H23B	109.5
C2—C1—H1A	110.1	C18—C23—H23C	109.5
C2—C1—H1B	110.1	H23A—C23—H23B	109.5
C2—N2—C3	111.46 (12)	H23A—C23—H23C	109.5
C2—N2—C7	106.21 (12)	H23B—C23—H23C	109.5
C5—N2—C2	110.78 (13)	C19—C24—H24A	109.5
C5—N2—C3	106.66 (13)	C19—C24—H24B	109.5
C5—N2—C7	110.95 (12)	C19—C24—H24C	109.5
C7—N2—C3	110.85 (13)	H24A—C24—H24B	109.5
C1—C2—H2A	107.8	H24A—C24—H24C	109.5
C1—C2—H2B	107.8	H24B—C24—H24C	109.5
N2—C2—C1	117.89 (14)	C19—C25—H25A	109.5
N2—C2—H2A	107.8	C19—C25—H25B	109.5
N2—C2—H2B	107.8	C19—C25—H25C	109.5
H2A—C2—H2B	107.2	H25A—C25—H25B	109.5
N2—C3—H3A	108.5	H25A—C25—H25C	109.5
N2—C3—H3B	108.5	H25B—C25—H25C	109.5
H3A—C3—H3B	107.5	C16'—C11'—S1	123.9 (5)
C4—C3—N2	115.21 (15)	C16'—C11'—C12'	119.4 (5)
C4—C3—H3A	108.5	C12'—C11'—S1	116.3 (4)
C4—C3—H3B	108.5	C11'—C16'—C15'	118.7 (6)
C3—C4—H4A	109.5	C11'—C16'—C18'	124.9 (6)
C3—C4—H4B	109.5	C15'—C16'—C18'	116.1 (5)
C3—C4—H4C	109.5	C11'—C12'—C17'	124.8 (4)
H4A—C4—H4B	109.5	C13'—C12'—C11'	118.9 (4)
H4A—C4—H4C	109.5	C13'—C12'—C17'	115.8 (3)
H4B—C4—H4C	109.5	C12'—C13'—H13'	118.9
N2—C5—H5A	108.6	C12'—C13'—C14'	122.1 (3)
N2—C5—H5B	108.6	C14'—C13'—H13'	118.9
H5A—C5—H5B	107.6	C13'—C14'—C19'	121.6 (3)
C6—C5—N2	114.60 (14)	C15'—C14'—C13'	117.1 (3)
C6—C5—H5A	108.6	C15'—C14'—C19'	121.1 (3)
C6—C5—H5B	108.6	C16'—C15'—H15'	118.5
C5—C6—H6A	109.5	C14'—C15'—C16'	122.9 (5)
C5—C6—H6B	109.5	C14'—C15'—H15'	118.5
C5—C6—H6C	109.5	C12'—C17'—H17'	108.2
H6A—C6—H6B	109.5	C12'—C17'—C20'	112.1 (3)
H6A—C6—H6C	109.5	C20'—C17'—H17'	108.2
H6B—C6—H6C	109.5	C21'—C17'—C12'	108.5 (3)
N2—C7—H7A	108.5	C21'—C17'—H17'	108.2
N2—C7—H7B	108.5	C21'—C17'—C20'	111.6 (3)
H7A—C7—H7B	107.5	C16'—C18'—H18'	108.1
C8—C7—N2	115.17 (15)	C22'—C18'—C16'	110.1 (4)
C8—C7—H7A	108.5	C22'—C18'—H18'	108.1
C8—C7—H7B	108.5	C23'—C18'—C16'	112.1 (5)
C7—C8—H8A	109.5	C23'—C18'—H18'	108.1
C7—C8—H8B	109.5	C23'—C18'—C22'	110.3 (4)
C7—C8—H8C	109.5	C14'—C19'—H19'	107.6
H8A—C8—H8B	109.5	C14'—C19'—C24'	110.1 (4)
H8A—C8—H8C	109.5	C14'—C19'—C25'	113.0 (3)
H8B—C8—H8C	109.5	C24'—C19'—H19'	107.6
C12—C11—S1	119.8 (4)	C25'—C19'—H19'	107.6
C16—C11—S1	121.0 (4)	C25'—C19'—C24'	110.8 (5)
C16—C11—C12	118.9 (5)	C17'—C20'—H20D	109.5
C11—C12—C17	123.3 (4)	C17'—C20'—H20E	109.5
C13—C12—C11	119.7 (4)	C17'—C20'—H20F	109.5
C13—C12—C17	116.9 (3)	H20D—C20'—H20E	109.5
C12—C13—H13	118.7	H20D—C20'—H20F	109.5
C14—C13—C12	122.5 (3)	H20E—C20'—H20F	109.5
C14—C13—H13	118.7	C17'—C21'—H21D	109.5
C13—C14—C15	116.5 (4)	C17'—C21'—H21E	109.5
C13—C14—C19	123.6 (3)	C17'—C21'—H21F	109.5
C15—C14—C19	119.8 (4)	H21D—C21'—H21E	109.5
C14—C15—H15	117.8	H21D—C21'—H21F	109.5
C14—C15—C16	124.5 (5)	H21E—C21'—H21F	109.5
C16—C15—H15	117.8	C18'—C22'—H22D	109.5
C11—C16—C18	126.4 (5)	C18'—C22'—H22E	109.5
C15—C16—C11	117.6 (5)	C18'—C22'—H22F	109.5
C15—C16—C18	115.9 (5)	H22D—C22'—H22E	109.5
C12—C17—H17	108.0	H22D—C22'—H22F	109.5
C20—C17—C12	109.2 (3)	H22E—C22'—H22F	109.5
C20—C17—H17	108.0	C18'—C23'—H23D	109.5
C20—C17—C21	112.3 (3)	C18'—C23'—H23E	109.5
C21—C17—C12	111.3 (3)	C18'—C23'—H23F	109.5
C21—C17—H17	108.0	H23D—C23'—H23E	109.5
C16—C18—H18	107.1	H23D—C23'—H23F	109.5
C16—C18—C23	112.2 (4)	H23E—C23'—H23F	109.5
C22—C18—C16	111.4 (4)	C19'—C24'—H24D	109.5
C22—C18—H18	107.1	C19'—C24'—H24E	109.5
C22—C18—C23	111.6 (3)	C19'—C24'—H24F	109.5
C23—C18—H18	107.1	H24D—C24'—H24E	109.5
C14—C19—H19	107.6	H24D—C24'—H24F	109.5
C24—C19—C14	114.7 (3)	H24E—C24'—H24F	109.5
C24—C19—H19	107.6	C19'—C25'—H25D	109.5
C24—C19—C25	109.8 (4)	C19'—C25'—H25E	109.5
C25—C19—C14	109.3 (3)	C19'—C25'—H25F	109.5
C25—C19—H19	107.6	H25D—C25'—H25E	109.5
C17—C20—H20A	109.5	H25D—C25'—H25F	109.5
C17—C20—H20B	109.5	H25E—C25'—H25F	109.5
			
S1—N1—C1—C2	94.86 (14)	C13—C12—C17—C20	60.6 (4)
S1—C11—C12—C13	170.0 (3)	C13—C12—C17—C21	−63.9 (4)
S1—C11—C12—C17	−13.0 (6)	C13—C14—C15—C16	0.0 (7)
S1—C11—C16—C15	−168.3 (4)	C13—C14—C19—C24	14.0 (5)
S1—C11—C16—C18	13.7 (8)	C13—C14—C19—C25	−109.8 (4)
S1—C11'—C16'—C15'	163.2 (4)	C14—C15—C16—C11	−3.9 (9)
S1—C11'—C16'—C18'	−22.9 (8)	C14—C15—C16—C18	174.2 (4)
S1—C11'—C12'—C13'	−164.4 (3)	C15—C14—C19—C24	−170.1 (4)
S1—C11'—C12'—C17'	23.3 (5)	C15—C14—C19—C25	66.1 (5)
O1—S1—N1—C1	−50.19 (14)	C15—C16—C18—C22	75.9 (6)
O1—S1—C11—C12	−166.8 (3)	C15—C16—C18—C23	−50.0 (6)
O1—S1—C11—C16	7.5 (5)	C16—C11—C12—C13	−4.3 (6)
O2—S1—N1—C1	−176.69 (12)	C16—C11—C12—C17	172.6 (4)
O2—S1—C11—C12	−44.1 (4)	C17—C12—C13—C14	−176.8 (3)
O2—S1—C11—C16	130.1 (4)	C19—C14—C15—C16	−176.3 (5)
N1—S1—C11—C12	70.0 (4)	C11'—S1—N1—C1	64.1 (2)
N1—S1—C11—C16	−115.7 (5)	C11'—C16'—C15'—C14'	1.7 (8)
N1—C1—C2—N2	179.67 (13)	C11'—C16'—C18'—C22'	−120.3 (6)
C2—N2—C3—C4	60.00 (19)	C11'—C16'—C18'—C23'	116.5 (6)
C2—N2—C5—C6	−62.17 (18)	C11'—C12'—C13'—C14'	−0.6 (5)
C2—N2—C7—C8	176.93 (14)	C11'—C12'—C17'—C20'	−129.7 (4)
C3—N2—C2—C1	61.08 (19)	C11'—C12'—C17'—C21'	106.5 (4)
C3—N2—C5—C6	176.37 (15)	C16'—C11'—C12'—C13'	8.1 (6)
C3—N2—C7—C8	−61.84 (18)	C16'—C11'—C12'—C17'	−164.2 (4)
C5—N2—C2—C1	−57.52 (18)	C12'—C11'—C16'—C15'	−8.7 (8)
C5—N2—C3—C4	−178.96 (15)	C12'—C11'—C16'—C18'	165.2 (5)
C5—N2—C7—C8	56.47 (19)	C12'—C13'—C14'—C15'	−6.2 (5)
C7—N2—C2—C1	−178.09 (14)	C12'—C13'—C14'—C19'	178.9 (3)
C7—N2—C3—C4	−58.08 (19)	C13'—C12'—C17'—C20'	57.7 (4)
C7—N2—C5—C6	55.54 (19)	C13'—C12'—C17'—C21'	−66.0 (4)
C11—S1—N1—C1	71.5 (2)	C13'—C14'—C15'—C16'	5.6 (7)
C11—C12—C13—C14	0.3 (5)	C13'—C14'—C19'—C24'	75.7 (5)
C11—C12—C17—C20	−116.4 (4)	C13'—C14'—C19'—C25'	−48.8 (5)
C11—C12—C17—C21	119.1 (4)	C15'—C16'—C18'—C22'	53.7 (6)
C11—C16—C18—C22	−106.2 (6)	C15'—C16'—C18'—C23'	−69.5 (6)
C11—C16—C18—C23	127.9 (6)	C15'—C14'—C19'—C24'	−99.0 (5)
C12—C11—C16—C15	6.0 (8)	C15'—C14'—C19'—C25'	136.5 (4)
C12—C11—C16—C18	−172.0 (4)	C17'—C12'—C13'—C14'	172.4 (3)
C12—C13—C14—C15	1.9 (5)	C18'—C16'—C15'—C14'	−172.7 (4)
C12—C13—C14—C19	178.0 (3)	C19'—C14'—C15'—C16'	−179.4 (5)

### Hydrogen-bond geometry (Å, º)

**Table:**

*D*—H···*A*	*D*—H	H···*A*	*D*···*A*	*D*—H···*A*
O4—H4*D*···O3	0.83 (2)	2.04 (2)	2.867 (2)	171 (2)
O3—H3*C*···O5	0.90 (2)	1.83 (2)	2.725 (2)	174 (2)
O3—H3*D*···O6^i^	0.85 (3)	2.08 (3)	2.912 (2)	169 (2)
O5—H5*C*···O2^ii^	0.83 (3)	2.09 (3)	2.901 (2)	165 (2)
O6—H6*D*···O4	0.86 (2)	1.95 (2)	2.787 (2)	167 (2)
O6—H6*E*···O3^iii^	0.82 (3)	2.03 (3)	2.845 (2)	170 (2)
O5—H5*D*···N1	0.84 (3)	2.05 (3)	2.881 (2)	170 (2)
O4—H4*E*···N1^ii^	0.92 (3)	2.06 (3)	2.959 (2)	165 (3)

Symmetry codes: (i) *x*−1, *y*, *z*; (ii) *x*+1, *y*, *z*; (iii) −*x*+3, −*y*+2, −*z*+1.
